# Ultra-Early and Early Changes in Bile Acids and Insulin after Sleeve Gastrectomy among Obese Patients

**DOI:** 10.3390/medicina55120757

**Published:** 2019-11-22

**Authors:** Adriana Florinela Cӑtoi, Alina Elena Pârvu, Aurel Mironiuc, Horațiu Silaghi, Ioana Delia Pop, Andra Diana Andreicuț

**Affiliations:** 1 Department of Pathophysiology, Faculty of Medicine, “Iuliu Hațieganu” University of Medicine and Pharmacy, 400012 Cluj-Napoca, Romania; parvualinaelena@umfcluj.ro (A.E.P.); cecan.andra@umfcluj.ro (A.D.A.); 2 2nd Surgical Clinic, Department of Surgery, Faculty of Medicine, “Iuliu Hațieganu” University of Medicine and Pharmacy, 400012 Cluj-Napoca, Romania; aurel.mironiuc@umfcluj.ro; 3 5th Surgical Clinic, Department of Surgery, Faculty of Medicine, “Iuliu Hațieganu” University of Medicine and Pharmacy, 400012 Cluj-Napoca, Romania; horatiu.silaghi@umfcluj.ro; 4 Department of Exact Sciences, University of Agricultural Sciences and Veterinary Medicine Cluj-Napoca, 400372 Cluj-Napoca, Romania; popioana@usamvcluj.ro

**Keywords:** severe obesity, bile acids, insulin resistance, sleeve gastrectomy

## Abstract

*Background and Objective:* In obese patients, sleeve gastrectomy (SG) has shown mixed results on bile acid (BA) values. The aim of our study was to examine the potential ultra-early and early changes of the circulating total BA in relation with the changes of insulin resistance (IR) in obese patients submitted to laparoscopic SG. *Materials and Methods:* Twenty-four obese subjects were investigated for body mass index (BMI), total fasting BA, insulin, homeostasis model assessment of insulin resistance (HOMA-IR), and leptin before and at 7 and 30 d after SG. *Results:* After surgery, mean BMI decreased at the first (*p* < 0.001) and at the second time point (*p* < 0.001) relative to baseline. Total fasting BA values did not change significantly at 7 d (*p* = 0.938) and at 30 d (*p* = 0.289) after SG. No significant changes were found at 7 d (*p* = 0.194, *p* = 0.34) and 30 d (*p* = 0.329, *p* = 0.151) after surgery regarding fasting insulin and HOMA-IR, respectively. However, a trend of increased total fasting BA and decreased fasting insulin and HOMA- after laparoscopic SG has been found. Negative correlations between total fasting BA and insulin (r = −0.807, *p* = 0.009), HOMA-IR (r = −0.855, *p* = 0.014), and blood glucose (r = −0.761, *p* = 0.047), respectively, were observed at one month after SG. *Conclusion:* In conclusion, here, we found a lack of significant changes in total fasting BA, insulin, and HOMA-IR ultra-early and early after SG, which precluded us to consider a possible relation between the variations of BA and IR. However, the presence of the tendency for total fasting BA to increase and for insulin and HOMA-IR to decrease, as well as of the negative correlations one month after laparoscopic SG, suggest that this surgery brings about some changes that point towards the existence, and possibly towards the restoration, at least to some extent, of the link between BA and glucose metabolism.

## 1. Introduction

Obesity has increased worldwide at an alarming rate [1]. Beyond the excess of fat mass, obese people display a gamut of disturbances such as type 2 diabetes mellitus (T2DM), dyslipidemia, and hypertension that impair health and reduce lifespan [2]. Among people with severe obesity (BMI ≥ 35 kg/m^2^), three out of four will present, to some extent, insulin resistance (IR), which is the cornerstone for several obesity-related diseases as previously described [3,4,5].

For patients with severe obesity (BMI ≥ 40 kg/m^2^, or ≥ 35 kg/m^2^ with associated diseases) significant weight loss is commonly hampered, making bariatric surgery the adequate means to attain this goal and to further restore metabolic health [6]. Indeed, apart from weight reduction, the various procedures of bariatric surgery have proven to be beneficial in inducing important metabolic and hormonal effects, out of which, noteworthily, some seem to be independent of the amount of fat loss [7,8,9]. In this respect, the resolution of IR and T2DM are often observed as early as a few days after bariatric surgery, suggesting that these improvements do not go in parallel with the gradual changes in body weight [7,10,11]. Several mechanisms have been proposed for such metabolic benefits, and among them, bile acids (BAs) have gained momentum [7,9,11].

Emerging data have shown that BAs work as key regulator molecules of glucose and lipid metabolism that act via binding to membrane or nuclear receptors [9,10,11,12]. In severely obese individuals, strong evidence has revealed that the same organs that display impaired insulin signaling are also targeted by BA, leading to significant disruptions in BA physiology [3]. In fact, BA alterations have been depicted in obesity, IR, and T2DM [13,14,15,16]. More precisely, BA levels have been reported to be low in subjects with obesity, while the presence of T2DM seems to induce an increase of these values as compared to obesity alone [14,17,18,19]. Given the effect of BA on metabolic functions, they are regarded as a possible therapeutic target in T2DM and obesity treatments [20]. In morbidly obese, prior to bariatric surgery, ursodeoxycholic acid has been demonstrated to induce neutral lipid accumulation in both liver and visceral white adipose tissue [21]. Also, BA sequestrants are involved in increasing insulin sensitivity and seem to reduce appetite in healthy humans; thus, they can be considered as a therapeutic strategy for weight loss [22,23].

Overall, bariatric surgery by both Roux-en-Y gastric bypass (RYGB) and sleeve gastrectomy (SG), the most performed techniques at the moment, has been reported to induce important changes in BA metabolism related or nonrelated to weight loss [24,25]. These changes seem to promote metabolic health via improving insulin signaling and reducing IR, thus making, once more, BA an important therapeutic target [3,12,25]. However, while circulating BAs have been demonstrated to increase following RYGB [13,18,26,27,28], the effect of SG on BA has shown mixed results. Namely, some authors reported increased concentrations [13,29,30], others decreased [31] circulating levels of BA, and finally, others detected no change after SG [32,33]. Most importantly, the exact timing of the BA changes and their potential role in the reduction of IR and restauration of insulin sensitivity are not fully elucidated [3,11,34]. Therefore, the present study sought to test the hypothesis of ultra-early and early changes of the circulating total BA as well as of the existence of a relation between these changes and the modifications of IR in severely obese patients submitted to laparoscopic SG. In order to do this, we investigated the levels of total fasting BA, fasting insulin, homeostasis model assessment of insulin resistance (HOMA-IR), and fasting blood glucose in severely obese patients at 7 and 30 d after laparoscopic SG.

## 2. Materials and Methods

### 2.1. Patients and Study Design

The present study enrolled 24 obese patients (15 female and 9 male) aged between 31 and 61 years, selected form the Second Surgical Clinic from Cluj-Napoca, Romania, between 2014 and 2015. They all met the 1991 National Institute of Health (NIH) Consensus Conference Guidelines for bariatric procedures (BMI ≥ 40 kg/m^2^ or BMI 35–40 kg/m^2^ with at least one comorbidity) and were submitted to laparoscopic SG. Patients with T2DM treated with insulin or other severe endocrine diseases, gallbladder stones, previous cholecystectomy, gut resection, inflammatory and infectious diseases, as well as alcoholism and major psychiatric diseases were excluded from the study.

The obese patients were evaluated before and after laparoscopic SG at 7 and at 30 d respectively. The protocol consisted in measurements of weight and height and withdrawal of blood samples for the evaluation of total BA, insulin, blood glucose, as well as leptin and lipids at each time point. Blood samples were collected in the morning after overnight fasting and underwent centrifugation in order to obtain serum. The serum samples were run immediately or otherwise stored at −80 °C until analysis.

The study was approved by the Ethics Committee of the “Iuliu Hațieganu” University of Medicine and Pharmacy from Cluj-Napoca (503/15.12.2011, 19.01.2012, 341/2.06.2015) and followed the ethical principles of the Helsinki Declaration. All patients were provided details about the research, and informed consent for inclusion was given by all of them.

### 2.2. Study Measurements

BMI was calculated according to the ratio between weight (kg)/squared height (m^2^), and the percent of excess BMI loss (%EBMIL) was calculated by using the %EBMIL = ((initial BMI − postop BMI)/(initial BMI − 25)) × 100 formula [35]. The evaluation of IR was performed by the homeostasis model assessment of insulin resistance (HOMA-IR) using the following formula: HOMA-IR = (fasting insulin µIU/mL × fasting glucose mg/dL)/22.5 × 18 [36]. We utilized the Friedewald formula in order to calculate the low-density lipoprotein cholesterol (LDL-C) concentration.

The measurements of serum fasting insulin and leptin were realized using commercial ELISA kits from DIAsource ImmunoAssays (KAP1251 and KAP2281 respectively, Louvain-la-Neuve, Belgium), while total fasting BA values were determined using the ELISA kit form Abbexa (abx052582, Cambridge, UK) according to the specific protocol of the manufacturer. Fasting blood glucose, total cholesterol, high-density cholesterol (HDL-C), and triglycerides were measured by the standard enzymatic colorimetric methods on an automatic analyzer.

### 2.3. Surgical Procedure

The performed surgical procedure (i.e., laparoscopic SG) has been described elsewhere [37].

### 2.4. Statistical Analysis

Continuous variables with normal probability distributions were expressed using descriptive statistics as mean ± standard deviation. Variables that deviated from the normal distribution were described by interquartile intervals (Q_1_; Q_3_), where Q_1_ is the first quartile and Q_3_ is the third quartile. We used the paired Student t-test or Wilcoxon test to analyze the changes in distributions of the studied characteristics at every time point after surgery. Bivariate, linear correlations between total fasting BA and metabolic variables were tested by Pearson’s correlation coefficient. For paired Student or Wilcoxon tests, we used Bonferroni’s correction in order to keep the error rate (α) to the specified level of 0.05, so statistical significance was achieved when the *p*-value < 0.025. In all tests, pairwise deletion was applied because there was missing data due to the different number of subjects at every follow-up point. Statistical analyses were performed with IBM SPSS v.19 (IBM Corp, Armonk, NY, USA) and StatSoft Inc., 1984–2004. Statistica for Windows (Software-system for data-analyses), Version 7.0. Tulsa, OK, USA.

## 3. Results

**Figure 1:**
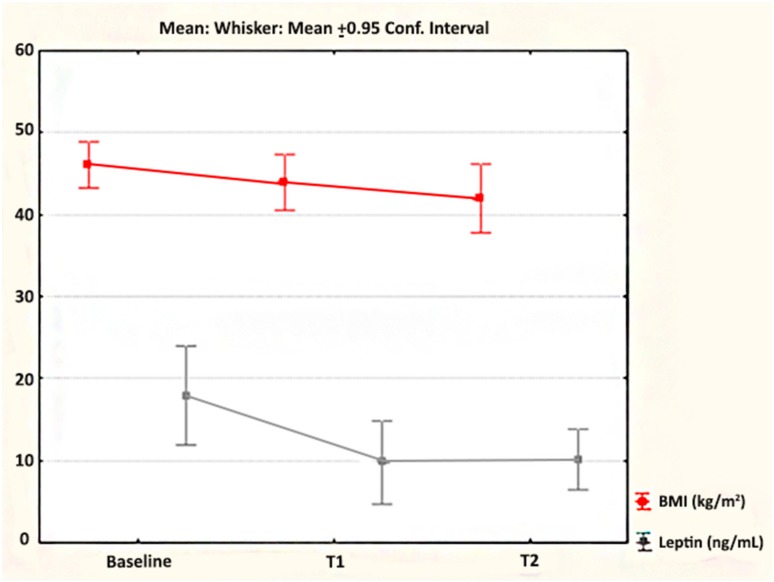
The dynamics of BMI (body mass index) and leptin after laparoscopic SG (sleeve gastrectomy).

After laparoscopic SG, we found statistically significant differences in the mean BMI values at the 7th day after operation (*p* < 0.001) and at the 30th day after operation (*p* < 0.001) relative to baseline (Figure 1). BMI changes revealed an estimated mean reduction of 2.65 kg/m^2^ (95% CI: 2.04, 3.26) at 7 d and of 5.45 kg/m^2^ (95% CI: 3.77, 7.13) at 30 d after laparoscopic SG. The %EBMIL was 13.23% ± 7.59% at 7 d and 26.23% ± 13.98% at 30 d after surgery. The mean leptin levels were statistically different at the first (7th d) (*p* = 0.001) and at the second time point (30th d) (*p* = 0.006) after surgery (Figure 1). Estimated mean reductions of 10.71 ng/mL (95% CI: 5.23, 16.19) at 7 d and of 6.81 ng/mL (95% CI: 2.30, 11.33) at 30 d after laparoscopic SG were detected for leptin (Table 1).

**Figure 2:**
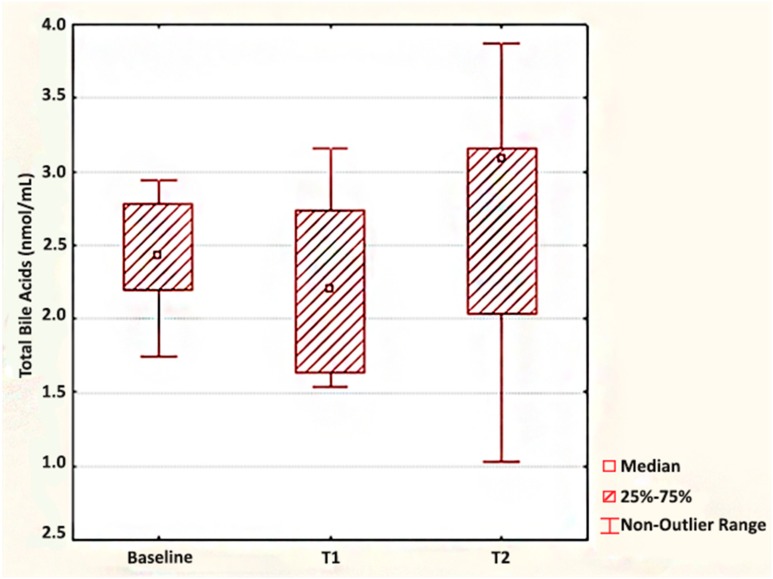
The dynamics of total fasting BA (bile acid) after laparoscopic SG.

With regard to total fasting BA values, we found no statistical changes at 7 d (*p* = 0.938) and at 30 d (*p* = 0.289) after surgery (Figure 2). For analyzed cases, we obtained estimated mean increases of BA values by the first time point (sum of negative versus positive rank values: 13 versus 15) and by the second time point (sum of negative versus positive rank values: 10 versus 26) (Table 1).

**Figure 3:**
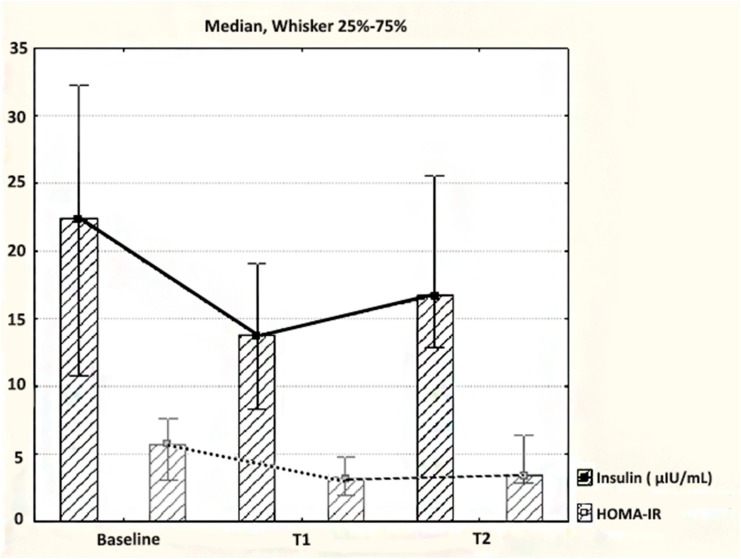
The dynamics of insulin and HOMA-IR after laparoscopic SG.

Although we did notice a different distribution of fasting insulin and HOMA-IR values at baseline as compared to both postsurgical follow up time points, we found no statistical differences by the 7th d (*p* = 0.194 for insulin and *p* = 0.34 for HOMA-IR) and by the 30th d after operation (*p* = 0.329 for insulin, *p* = 0.151 for HOMA-IR) (Figure 3). The analysis of fasting insulin and HOMA-IR changes, as compared to the baseline moment, revealed reductions of the values at the 7th d (sum of negative versus positive rank values: 74 versus 31 for fasting insulin and 60 versus 31 for HOMA-IR) and at the 30th d after surgery (sum of rank values: 98 versus 55 for fasting insulin and 86 versus 34 for HOMA-IR). We found no statistically significant changes for fasting glucose at 7 d (*p* = 0.707) and at 30 d after SG (*p* = 0.387) (Table 1).

No statistically significant differences were found for total cholesterol and LDL cholesterol at the first time point (*p* = 0.692 and *p* = 0.421). Also, a lack of significance was noticed at the second time point after surgery for total cholesterol (*p* = 0.129). However, we observed a significant change for LDL cholesterol (*p* = 0.024) at 30 d after laparoscopic SG. With respect to triglycerides, there were no significant changes at 7 d (*p* = 0.89) and at 30 d (*p* = 0.84) after surgery. Also, no significant change was found for HDL cholesterol at 7 d (*p* = 0.78), whereas statistical significance was detected at 1 month after laparoscopic SG (*p* = 0.008) (Table 1).

### Correlations

We did not detect any significant correlations at baseline and at 1 week after laparoscopic SG between fasting total BA and the other parameters. However, at one month after surgery, we observed significant, negative correlations between fasting total BA and insulin (r = −0.807, *p* = 0.009), HOMA-IR (r = −0.855, *p* = 0.014), and blood glucose (r = −0.761, *p* = 0.047), respectively (Table 2).

## 4. Discussion

The precise mechanisms behind the weight-independent effects of bariatric surgery involved in IR remain poorly understood [38,39]. BAs have been demonstrated to be altered in obesity and T2DM, and the role of SG in changing their metabolism has been discussed [13,14,15]. In our study, we investigated the ultra-early and early changes of fasting total BA in relation with the changes of fasting insulin and HOMA-IR, respectively, in severely obese patients following laparoscopic SG (7 and 30 d after surgery). We hypothesized that potential ultra-early and early variations of fasting total BA might be related to IR modifications. Here, although we identified a trend of fasting total BA to increase and of fasting insulin and HOMA-IR to decrease after surgery, these changes were not statistically significant. At one month after SG, we observed significant, negative correlations between fasting total BA and insulin, HOMA-IR, and blood glucose, respectively.

The relationship of BA with IR in obesity is complex [40]. Alterations in total BA have been shown in subjects with prediabetes, T2DM, and IR [41]. In a cross-sectional study that included 9603 subjects, Sun et al. [42] showed an increase of BA in IR individuals regardless of the presence/absence of T2DM, and they pointed out the important role of IR in BA metabolism regulation. However, noteworthily, the study enrolled subjects that were not severely obese. De Vuono et al. [43] investigated candidates for SG (BMI of 45 ± 7kg/m^2^) and found higher levels of both primary and secondary BA levels in IR than in non-IR obese before undergoing surgery. The authors reported that BA values were positively associated with the HOMA-IR index, which unexpectedly was not found in our study group. Furthermore, a lack of correlation between BA and BMI values was noticed in our study, which is in line with the finding of De Vuono et al. [43] and with other reports where obese subjects showed lower circulating BA levels as compared to normal-weight individuals [13,17,18]. Also, obesity is associated with nonalcoholic fatty liver disease (NAFLD), a condition that is accompanied by a decrease in the hepatic activity of peroxisome proliferator-activated receptor alpha (PPAR-alpha), which, in turn, is directly related to HOMA-IR and increases the pro-lipogenic status of the liver [44,45]. Most importantly, it has been revealed that PPAR-alpha holds an essential role in regulating BA synthesis and signaling [46].

Emerging data have shown that BAs are signaling molecules, acting on receptors such as the nuclear receptor farnesoid X receptor (FXR) and membrane Takeda G protein-coupled receptor-5 (TGR5), through which they are involved in the regulation of glucose, lipid, and energy, metabolism [47,48,49]. Once in the intestine, the BAs bind to FXR and stimulate the synthesis of fibroblast growth factor 19 (FGF19), which acts in an endocrine manner at the liver level to downregulate cholesterol *7α-hydroxylase* (*CYP7A1*) gene transcription and, thus, to reduce the synthesis and secretion of BA [48]. Upon acting on TGR5, which is expressed in enteroendocrine cells, BAs increase glucagon-like peptide-1 (GLP-1) release and improve insulin secretion and sensitivity [9,50]. Finally, insulin seems to modulate BA synthesis, exerting inhibitory effects, by acting indirectly on the FXR and directly at the CYP7A1 level. In IR, some mechanisms work in concert. For example, insulin actions become blunted and along with increased blood glucose that, when present, upregulates FXR finally lead to dysregulation of BA synthesis [16,51]. On the other hand, Watanabe et al. [52] showed that oral administration of BA diminishes weight gain and IR and increases energy expenditure in animal models.

Several human studies have reported total BAs change after bariatric surgery [53]. Overall, the variations consist of increased circulating values, mainly after RYGB [13,14,18,19,26,27,28,29,54,55,56,57].

Following SG, data are less consistent. Some researchers showed increased [13,29,30] or decreased [31] levels, whereas others showed transiently decreased [40] or unchanged [17,32,33] levels at different time points after surgery associated, or not, with metabolic improvements (Table 3). Still, the question arises if there is an acute change at one week and one month after surgery and if this change is related or not to the improvement of IR. Herein, although we identified a trend of total fasting BA to increase and of fasting insulin and HOMA-IR, respectively, to decrease after SG, the changes were not statistically significant. Similar results were reported by Shimizu et al. [32] who showed no changes of BA levels at one and six months after SG, but with no evaluation of insulin sensitivity and production, as four of the patients with T2DM were receiving insulin therapy. In keeping with the trend of total BA increase reported by us, Chen et al. [34] showed an acute, significant elevation in total and almost all BA species after both gastric bypass and SG, which was seen as early as three days and was sustained three months after surgery. Also, Jahansouz et al. [29] pointed out that an increase in serum BA levels after SG, and a trend toward increased levels with RYGB, occurred acutely at one week after surgery. Noteworthily, this increase is unrelated to hypocaloric restriction [29]. On the other hand, Escalona et al. [40] reported a drop in BA levels at one month after SG (which, however, increased later after SG), whereas Nakatami et al. [19] showed rising total BA at one and three months after both malabsorptive procedures (RYGB and SG with duodenujejunal bypass) and restrictive procedures (SG and adjustable gastric banding). Also, insulin and HOMA-IR significantly decreased three months after malabsorptive procedures but not after restrictive procedures [19].

The exact mechanisms involved in BA elevations after bariatric surgery are not fully elucidated [12]. An accelerated nutrient transit to the distal small intestine, increased hepatic synthesis and/or altered enterohepatic recirculation of bile, as well as rearrangements of the intestinal gut microbiota, which are responsible for secondary BA formation, are some of the putative factors responsible for inducing postsurgical BA changes [6].

A relationship between increases in BA and metabolic improvement was reported by Chen et al. [34] as early as three days after surgery. These correlations regard improvement in insulin sensitivity as assessed by the Stumvoll Insulin Sensitivity Index (ISI), calculated using oral glucose tolerance test (OGTT) values, and not by HOMA-IR, which uses fasting glucose and insulin values. HOMA-IR seems to be less influenced by bariatric surgery than Stumvoll ISI [34]. While in our present study, we observed no significant changes in fasting insulin and HOMA-IR (although a decreasing trend was noticed after surgery), in a previous study, we found a significant reduction of HOMA-IR one month after SG [37]. The disparity between the results could be, most likely, explained by the basic characteristics of the selected patients [37]. SG is more than just a gastric restrictive procedure and, other than weight-loss-related mechanisms, may induce IR changes following SG, mechanisms that still need to be better explained [58,59]. Herein, although we could not evaluate the correlations between the changes of fasting total BA and insulin and HOMA-IR, respectively, because of the lack of significant changes, we did notice the presence of significant, negative correlations between fasting total BA and insulin, HOMA-IR, and blood glucose, respectively, one month after laparoscopic SG. Based on these findings, and also on the previously explained trend of these parameters (i.e., increase of fasting total BA and decrease of fasting insulin and HOMA-IR), we may argue that SG induces some modifications, which point towards the existence and, at least to some extent, the restoration of the link between BA and glucose metabolism. On the other hand, Steinert et al. [13] reported decreased plasma values of total basal BA at one week and a small, but not significant, increase at three months after both RYGB and SG. Also, an improvement in fasting insulin and HOMA-IR was observed as early as three months after surgery. The authors explained it is more likely that BA may contribute to metabolic improvements later after surgery [13]. Another two studies (human and animal) reported increased circulating BA after RYGB, but they pointed out that these changes are unlikely to contribute to early postsurgical improvement in glucose hemostasis [56,60].

In a very recent study, De Vuono et al. [43] reported a significant decrease of primary BA and increase of secondary BA one year after SG, changes that were independent of weight loss. Also, while primary BA reduction was positively correlated with IR amelioration, no association was found between secondary BA increase and IR parameter changes. The authors concluded that an involvement of BA changes in SG-induced IR and/or T2DM improvement or remission cannot be claimed. However, they argued the direct association between primary BA drop and IR amelioration suggest that IR is more likely to regulate primary BA synthesis, and not vice versa (i.e., BA does not influence glucose metabolism) [43].

One explanation for the differences between our results and the data from the literature might reside in the baseline characteristics of the patients. The presence/absence of T2DM and the degree of IR before surgery may influence BA changes after SG [28,56].

We acknowledge that the present study has some limitations. The small sample size and the presence of patients both with and without T2DM may have prevented us to detect important BA changes after surgery. Evaluation of the time trend for fasting total BA and other metabolic parameters is subject to restrictions because of the missing data, as the present study interpreted only the punctual changes of parameters at two time points after surgery as compared to baseline. Therefore, further studies are needed in order to evaluate the ascendant or descendant trends of these parameters. Further, more precise data could be obtained when evaluating all types of BA (primary and secondary) and the intestinal BA concentration. Finally, the pre- and postprandial measurements of BA might shed more light on the relationship between BA and blood glucose metabolism.

## 5. Conclusions

In the present study, we found a lack of significant changes in fasting total BA, insulin, and HOMA-IR ultra-early and early after SG, which precluded us to consider a possible relation between the variations of BA and IR. Nevertheless, the increasing trend of fasting total BA and the decreasing trend of insulin and HOMA-IR, together with the negative correlations found one month after laparoscopic SG, might suggest that this type of surgery induces some changes that point towards the existence and, possibly towards the restoration, at least to some extent, of the link between BA and glucose metabolism. Further studies are needed to confirm the post-SG variations and the connection between circulating BA and the improvement in insulin signaling, mostly during the early postoperative period.

## Figures and Tables

**Table 1:** Anthropometric and metabolic characteristics of the severely obese before and after laparoscopic SG (sleeve gastrectomy).

	Pre-SG (*n* = 24)	7 d after SG	30 d after SG
Weight (kg)	130.96 ± 26.66	126.94 ± 27.14 *	121.7 ± 29.46 *
BMI (kg/m^2^)	46.15 ± 6.70	43.98 ± 6.91 *	41.96 ± 7.48 *
Leptin (ng/mL)	17.89 ± 12.81	9.81 ± 9.48 *	10.15 ± 7.05 *
Fasting insulin(μIU/mL)	23.47 ± 13.20	13.71 (8.33–19.07)	16.69 (12.84–25.62)
Fasting glucose (mg/dL)	99.29 ± 21.57	96.88 ± 9.39	93.07 ± 10.24
Homeostasis model assessment of insulin resistance (HOMA-IR)	5.85 ± 3.62	3.19 (2.14–4.51)	3.40 (2.91–5.25)
Total fasting BA (nmol/mL)	2.45 ± 0.38	2.20 (1.64–2.74)	3.09 (2.03–3.16)
Total cholesterol (mg/dL)	195.58 ± 33.34	186.69 ± 23.47	184.47 ± 35.81
Triglycerides (mg/dL)	152 ± 63.62	116.50 (110–190.5)	144 (118.5–161.5)
High-density lipoprotein (HDL) cholesterol (mg/dL)	52.14 ± 13.88	76 (71–80)	76 (70.50–87.50) *
Low-density lipoprotein (LDL) cholesterol (mg/dL)	112.48 ± 40.11	94.33 ± 29.54	73.09 ± 29.25 *

The results are expressed as median and IQR (interquartile range) and as SD (standard deviation); * *p* < 0.025 (Bonferroni correction: alfa = 0.025, differences between baseline and each postoperative time point); the number values of anthropometric and metabolic parameters were different at each time point because of missing data (n at 7 d and 30 d were different, and *n* < 24).

**Table 2:** Correlations between fasting total BA and anthropometric and metabolic parameters.

Parameters	Total BA (nmol/mL) at Baseline		Total BA at 7 d after SG		Total BA at 30 d after SG	
	r	*p*	r	*p*	r	*p*
BMI (kg/m^2^)	−0.028	0.931	−0.071	0.879	0.145	0.756
Leptin (ng/mL)	−0.325	0.359	−0.293	0.444	−0.521	0.150
Fasting insulin (μIU/mL)	−0.232	0.467	−0.164	0.674	**−0.807**	**0.009**
Fasting glucose (mg/dL)	−0.370	0.236	−0.611	0.108	**−0.761**	**0.047**
HOMA-IR	−0.176	0.584	−0.180	0.670	**−0.855**	**0.014**
Total cholesterol (mg/dL)	0.000	1.000	−0.235	0.575	0.303	0.509
Triglycerides (mg/dL)	0.218	0.459	−0.347	0.399	0.837	0.019
HDL cholesterol (mg/dL)	-0.275	0.509	0.029	0.957	0.339	0.456
LDL cholesterol (mg/dL)	−0.096	0.821	0.240	0.568	0.218	0.638

**Table 3:** Changes in BA following SG.

Authors, Year of Publication	Sample Size	Postsurgical Follow-Up	Total Fasting BA Changes after Surgery
Steinert et al., 2013	7	1 week, 3 and 12 months	Decrease at one week Small but nonsignificant increase at 3 months Significant increase at one year
Haluzíková et al., 2013	17	6, 12, 24 months	No change
Escalona et al., 2016	19	1, 3, 6, 12 months	Decrease at one month Increase at 3, 6 and 12 months
Belgaumkar et al., 2016	18	6 months	No change
Jahansouz et al., 2016	12	7 d	Significant increase
Shimizu et al., 2017	10	1 and 6 months	No change
Chen et al., 2019	11	3 d and 3 months	Significant increase at 3 d Significant decrease at 3 months (but still higher than preoperative levels)
De Vuono et al., 2019	79	12 months	Significant reduction of primary BA and increase of secondary BA
Huang et al., 2019	18	3 and 12 months	Significant decrease at 3 and 12 months after surgery
